# Polyunsaturated Fatty Acid (PUFA) Status in Pregnant Women: Associations with Sleep Quality, Inflammation, and Length of Gestation

**DOI:** 10.1371/journal.pone.0148752

**Published:** 2016-02-09

**Authors:** Lisa M. Christian, Lisa M. Blair, Kyle Porter, Mary Lower, Rachel M. Cole, Martha A. Belury

**Affiliations:** 1 Department of Psychiatry & Behavioral Health, The Ohio State University Wexner Medical Center, Columbus, Ohio, United States of America; 2 The Institute for Behavioral Medicine Research, The Ohio State University Wexner Medical Center, Columbus, Ohio, United States of America; 3 Department of Obstetrics and Gynecology, The Ohio State University Wexner Medical Center, Columbus, Ohio, United States of America; 4 Department of Psychology, The Ohio State University, Columbus, Ohio, United States of America; 5 College of Nursing, The Ohio State University, Columbus, Ohio, United States of America; 6 Center for Biostatistics, The Ohio State University, Columbus, Ohio, United States of America; 7 Program of Nutrition in the Department of Human Sciences, The Ohio State University, Columbus, Ohio, United States of America; Max Delbrueck Center for Molecular Medicine, GERMANY

## Abstract

Mechanistic pathways linking maternal polyunsaturated fatty acid (PUFA) status with gestational length are poorly delineated. This study examined whether inflammation and sleep quality serve as mediators, focusing on the antiinflammatory ω-3 docosahexaenoic acid (DHA; 22:6n3) and proinflammatory ω-6 arachidonic acid (AA; 20:4n6). Pregnant women (*n* = 135) provided a blood sample and completed the Pittsburgh Sleep Quality Index (PSQI) at 20–27 weeks gestation. Red blood cell (RBC) fatty acid levels were determined by gas chromatography and serum inflammatory markers [interleukin (IL)-6, IL-8, tumor necrosis factor-α, IL-1β, and C-reactive protein] by electrochemiluminescence using high sensitivity kits. Both higher serum IL-8 (95% CI = 0.10,3.84) and poor sleep (95% CI = 0.03,0.28) served as significant mediators linking lower DHA:AA ratios with shorter gestation. Further, a serial mediation model moving from the DHA:AA ratio → sleep → IL-8 → length of gestation was statistically significant (95% CI = 0.02, 0.79). These relationships remained after adjusting for depressive symptoms, age, BMI, income, race, and smoking. No interactions with race were observed in relation to length of gestation as a continuous variable. However, a significant interaction between race and the DHA:AA ratio in predicting preterm birth was observed (p = 0.049); among African Americans only, odds of preterm birth decreased as DHA:AA increased (p = 0.048). These data support a role for both inflammatory pathways and sleep quality in linking less optimal RBC PUFA status with shorter gestation in African American and European American women and suggest that African-Americans have greater risk for preterm birth in the context of a low DHA:AA ratio.

## Introduction

It is well established that long chain polyunsaturated fatty acids (LC-PUFAs), particularly the ω-3 docosahexanoic acid (DHA; 22:6n-3), are critical to fetal growth, neural and retinal development. Reflecting transfer of fatty acids from the mother to the developing fetus, maternal plasma and red blood cell (RBC) DHA levels decrease markedly and progressively during normal pregnancy and may not return to normal until several months postpartum while infant DHA levels exceed those of the mother for an extended period, particularly among those who are breastfed [1–3]. This preferential transfer of fatty acids potentiates the likelihood of poor fatty acid status in women during pregnancy.

In addition to their importance for fetal development, maternal fatty acid consumption may influence length of gestation. However, this literature is mixed. Some meta-analyses show no effect of ω-3 supplementation on preterm birth [4–6]. Other meta-analyses of studies in low as well as high-risk populations indicate that maternal consumption of LC-PUFAs, particularly lower DHA, predicts shorter length of gestation and/or risk for preterm birth [7–10]. This effect is supported in a phase III, double-blind RCT of 350 women which demonstrated that supplementation with 600 mg/d of DHA in the last half of pregnancy resulted in longer gestation and greater infant size (birth weight, length, and head circumference)[11]. Although evidence in this regard is accumulating, at present there is not sufficient evidence to support the routine use of supplements during pregnancy in order to lengthen gestation. In addition, the mechanistic pathways potentially linking fatty acid consumption and length of gestation are poorly delineated.

A key potential pathway is inflammation. DHA has known antiinflammatory properties [12]. In contrast, although it also plays an important role in fetal growth and development, the ω-6 arachidonic acid (AA) produces proinflammatory mediators [12] which play a role in the initiation of delivery [13]. Whereas DHA levels are highly dependent on diet, AA levels are less affected by intake but instead influenced greatly by genetic factors [14]. Thus, a biologically plausible pathway by which dietary DHA could lengthen gestation is by dampening or counteracting the inflammatory effects of AA. In this manner, in addition to assessing DHA and AA status separately, the DHA:AA ratio may be a predictor of health outcomes.

An additional possible mechanistic pathway linking DHA consumption and length of gestation is effects on sleep. An estimated 22.1% of US adults meeting DSM-IV-TR criteria for insomnia, with significantly higher odds among women [15–17]. Pregnancy further exacerbates insomnia and other types of sleep disturbance [18–20]. Moreover, data from our own group and others links poor sleep in pregnancy with adverse outcomes including shorter gestation [21–25].

Observational and experimental evidence in animals as well as children and adults supports a role for LC-PUFAs in subjective sleep quality, objective sleep quality (per actigraphy), sleep architecture, and cardiovascular function during sleep [26–32]. Notably, sleep quality is linked with inflammation in a bidirectional manner [33–36] and thus could plausibly be induced by and/or exacerbate inflammatory effects of a low DHA:AA ratio. Despite unique vulnerabilities related to LC-PUFA depletion during pregnancy, whether sleep quality may play a mediating role linking fatty acids and length of gestation remains to be examined.

Our prior data demonstrate an association between poor sleep quality during pregnancy and risk for shorter gestation which was mediated by elevations in serum levels of the proinflammatory cytokine interleukin(IL)-8 [25]. In these prior analyses, effects were driven by African American women. In the current analyses, we build upon these findings to examine the role of fatty acid status within this model in the same sample of women. This study included 135 pregnant women assessed at mid-gestation. The current analyses examined whether RBC fatty acid status was linked to length of gestation via 1) serum inflammatory markers [interleukin (IL)-6, tumor necrosis factor (TNF)-α, IL-8, IL-1β, and C-reactive protein (CRP)] and 2) poor sleep quality as indexed by subjective reports on the Pittsburgh Sleep Quality Index. It was hypothesized that lower RBC DHA, higher AA, and a lower DHA:AA ratio would be linked to shorter gestation via these mediators. Given racial disparities in preterm birth, and our prior data demonstrating differential effects by race, we also examined effects of race within these models.

## Materials and Methods

### Study Design

These analyses utilized data from two concurrent studies examining different aspects of health and risk in pregnancy. Participants for both studies were recruited from The Ohio State University Wexner Medical Center and the surrounding community of Columbus, Ohio. Both studies were approved by The Ohio State University Biomedical Institutional Review Board. All participants completed written informed consent and privacy notifications and received modest compensation for participation. Study visits were conducted from 06/22/2009 through 06/24/2011.

The first study was a cross-sectional observational design which examined relationships among periodontal disease, race, and the effects of stress among 101 women from lower socioeconomic backgrounds (annual household income <$50,000) who were assessed in-person during the 2^nd^ trimester of pregnancy with birth outcome data obtained via medical record review. The second study examined 56 pregnant women in a longitudinal manner, with in-person assessments during each trimester of pregnancy and birth outcome data obtained via medical record review. For the current analyses, only data from the 2^nd^ trimester were included, as these corresponded to the timing of assessment of the first cohort. Thus, eight women who had missing data at the second trimester visit were excluded. In addition, 11 women participated in both protocols; their data were used only once. Fatty acid data was unavailable for one woman and two women had outlying values (≥ 5 SD beyond the mean) resulting in a final sample of 135 women.

Exclusion criteria for both samples included chronic health conditions or medications with implications for immune function (such as steroids), diagnosed fetal anomaly, antidepressant use, illicit drug use other than marijuana, or more than two alcoholic beverages per week during pregnancy (per self-report or medical record at time of enrollment). Any woman who reported acute illness (i.e. cold- or flu-like symptoms) or antibiotic use within 10 days was rescheduled.

### Demographics and Birth Outcomes

Age, race/ethnicity, education, annual household income, gravidity, and parity were collected by self-report. Pre-pregnancy body mass index (BMI; kg/m2) was calculated using self-reported pre-pregnancy weight and height measured at the first visit. Gestational age at delivery was determined by medical record review. This was utilized as a continuous variable, in addition, births were classified as full term (≥ 39 weeks), early term (37 weeks 0 days– 38 weeks 6 days), or preterm (<37 weeks) based on guidelines from ACOG [37].

### Measures of Sleep and Depressive Symptoms

Sleep quality was assessed by self-report using the Pittsburgh Sleep Quality Index (PSQI).[38] A score > 5 is indicative of clinically disturbed sleep. This measure includes seven subscales: subjective sleep quality, sleep latency (i.e., time to fall asleep), sleep duration, habitual sleep efficiency (i.e., time asleep/time in bed), sleep disturbance, use of sleeping medications, and daytime dysfunction. The PSQI has high diagnostic sensitivity and specificity in distinguishing good and poor sleepers.[38] Global scores as well as subscale scores show high test-retest reliability across short intervals in adults with insomnia.[39] The PSQI is commonly used in studies of pregnant women and has shown predictive validity for health outcomes in pregnancy as well as postpartum in prior studies.[40–42] In addition, the Center for Epidemiological Studies Depression Scale (CES-D) was used to assess depressive symptoms.[43] This is a 20-item measure with high internal consistency. Scores ≥ 16 indicate clinically significant depressive symptoms.

### Blood Samples

Blood samples were obtained via venipuncture. A 10 mL vacutainer collection tube was used to collect blood for cytokine assays. Serum was subsequently left to clot for 30 minutes at room temperature before being centrifuged at 1932g for 10 minutes. The serum was then aliquoted into 1ml cryovials. Each RBC PUFA sample was collected in a 6 mL EDTA tube on ice. The sample was centrifuged at 1932g for 10 minutes. The plasma was removed and the RBCs were stored in 1ml cryovials. All samples were stored at -80°C until the time of assay.

### Red Blood Cell Fatty Acid Assays

Lipids were extracted and methylated from erythrocytes using boron-trifluoride in methanol. Fatty acid methyl esters were analyzed by gas chromatography (Shimadzu, Columbia, MD) using a 30-m Omegawax 320 (Supelco-Sigma) capillary column. The helium flow rate was 30 ml/min and oven temperature ramped beginning at 175 C and held for 4 min then increased to 220 C at a rate of 3 C/min as previously described [44]. Retention times were compared to authentic standards for fatty acid methyl esters (Supelco-Sigma, St. Louis, MO and Matreya, Inc., Pleasant Gap, PA) and fatty acids are reported as percent of total identified. The intra-assay coefficient of variation was 11.1, 8.9%, and 20.3% for AA, DHA and EPA, respectively.

### Serum proinflammatory cytokines

Serum levels of interleukin (IL)-6, tumor necrosis factor (TNF)-α, IL-8, and IL-1β were assayed in duplicate with ultra-sensitive multiplex kits from Meso Scale Discovery (MSD) and chemilluminescence methodology using the Sector Imager 2400 (Meso Scale Discovery, 9238 Gaithers Rd., Gaithersburg, Md). Limits of detection were 0.61 pg/ml for IL-6, 2.4 pg/ml for TNF-α, 0.3 pg/ml for IL-8, and 0.61 pg/ml for IL-1β. All values were above the limits of detection. Intra-assay coefficients of variation (CVs) were between 5.21 to 10.66 and the inter-assay CVs were between 8.4 to 11.17. CRP values were determined using chemilluminescence methodology using the Immulite 1000 (Siemens Healthcare Diagnostics, Inc., 1717 Deerfield Rd., Deerfield, Il.) The limit of detection is 0.3 mg/L; all values were above the limit of detection. The intra and inter-assay coefficients of variation were 5.1% and 7.3%. All samples from the same participant were batched together and assayed on the same plate. Assays for the two studies were conducted at the same time and all plates were from the same lot.

### Statistical Methods

As specified in our hypotheses, analyses focused on AA and DHA. However, we also examined potential effects of eicosapentaenoic (EPA, 20:5n3) to ensure that expected effects of DHA were not better explained by levels of this other key form of ω-3 fatty acid. All statistical analyses were conducted using SAS/STAT software version 9.3 (Cary, NC). Baseline characteristics were compared between African American and European Americans by t-test, chi-square, or Fisher’s exact tests. Associations of RBC PUFAs with demographic variables were evaluated by t-test. Linear regression models were used to analyze the correlation between RBC PUFAs with length of gestation and inflammatory markers. Cytokine levels were log-transformed for analysis. Logistic regression models were fit to evaluate the associations between RBC PUFAs and preterm birth. Linear regression was used to evaluate the association of sleep quality with RBC PUFA. The total PSQI score was analyzed as a continuous variable. PSQI subscales are ordinal and were analyzed by cumulative logistic regression. All regression models adjusted for the covariates age, BMI, depressive symptoms, income, smoking status, and race. Also, in separate models, interactions between race and fatty acid levels were added to the logistic and linear regression models described above. Analyses were also performed separately by race. No adjustments were made for multiple comparisons.

Mediation effects for inflammation and sleep were analyzed using a regression based approach. This approach tests the indirect effect by multiplying the estimated coefficients for the regression models for the indirect path. A bootstrapping resampling method was used to estimate the indirect effect and construct corresponding 95% confidence intervals (Hayes, 2013). The advantage of this approach is that it makes no assumptions about the sampling distribution of the indirect effect. The mediation effect is significant if the 95% confidence interval does not include zero. The paths analyzed were from RBC PUFAs to IL-8 to length of gestation and from RBC PUFAs to sleep to length of gestation. A serial mediation analysis [45] from RBC PUFAs to sleep to IL-8 to length of gestation was also performed. All indirect mediating effects were evaluated using the PROCESS macro for SAS (http://www.processmacro.org).

## Results

### Sample Characteristics

Participants were 18 to 35 years of age (Mean = 23.8 ± 4.1) years. The sample was 60% African American and 65% reported an annual household income < $15,000. Additional sample characteristics are summarized in Table 1.

**Table 1:** Demographic Characteristics and RBC Fatty Acid levels.

	Total (n = 135)	African American (n = 78)	European American (n = 51)	Group comparison (p value)
**Age, mean (SD)**	23.8 (4.1)	23.9 (3.9)	23.4 (4.3)	0.47
**Body Mass Index, mean (SD)**	28.5 (8.1)	29.1 (8.0)	27.7 (8.6)	0.35
**Marital Status**				0.005
Married	22 (16.3%)	6 (7.7%)	14 (27.5%)	
Unmarried, in a relationship	80 (59.3%)	48 (61.5%)	29 (56.9%)	
Single	33 (24.4%)	24 (30.8%)	8 (15.7%)	
**Income**				0.011^a^
Less than $15,000	88 (65.2%)	56 (71.8%)	30 (58.8%)	
$15,000–$29,999	36 (26.7%)	20 (25.6%)	12 (32.5%)	
≥ $30,000	11 (8.1%)	2 (2.6%)	9 (17.7%)	
**Education**				0.21
High school or less	33 (24.4%)	22 (28.2%)	11 (21.6%)	
Some college	39 (28.9%)	26 (33.3%)	11 (21.6%)	
Bachelor’s degree	57 (42.2%)	27 (34.6%)	27 (52.9%)	
Some graduate school or higher	6 (4.4%)	3 (3.9%)	2 (3.9%)	
**Smoking**				0.036^b^
Current	18 (13.3%)	8 (10.3%)	10 (19.6%)	
Past	24 (17.8%)	9 (11.5%)	12 (23.5%)	
Never	93 (68.9%)	61 (78.2%)	29 (56.9%)	
**RBC Fatty Acid Levels**				
Docosahexaenoic Acid (22:6n3)	3.5 (0.7)	3.6 (0.7)	3.4 (0.8)	0.20
	1.9–5.8	2.2–5.6	1.9–5.8	
Eicosapentaenoic (20:5n3)	0.2 (0.1)	0.2 (0.1)	0.2 (0.1)	0.45
	0.1–0.7	0.1–0.7	0.1–0.5	
Arachidonic Acid (20:4n6)	14.5 (1.3)	14.9 (1.2)	14.0 (1.2)	<0.001
	10.5–17.7	12.2–17.7	10.5–16.7	
DHA:AA ratio	0.24 (0.05)	0.24 (0.05)	0.24 (0.06)	0.67
	0.15–0.46	0.15–0.37	0.15–0.46	

Note: Women of Hispanic ethnicity (n = 6) were excluded from comparisons by race. Fatty acid values are expressed as mg/100 mg of total fatty acids identified: mean (SD), range

^a^ A higher percentage of European Americans than African Americans had an income ≥ $30,000 (p = 0.003).

^b^ A higher percentage of African Americans than European Americans have never smoked (p = 0.010).

### Behavioral and Demographic Correlates of RBC PUFA levels

Age, BMI, smoking, and depressive symptoms were not associated with RBC AA or DHA. RBC AA levels were higher in African Americans than in European Americans (t(127) = 4.36, p < 0.001; Table 1). Also, compared to women with an annual household income < $15,000, those at $15,000 to $29,999 had higher RBC DHA (t(131) = 2.54, p = 0.012) and higher DHA:AA ratios (t(131) = 2.61, p = 0.010).

### RBC PUFAs and Serum Proinflammatory Markers

**Fig 1:**
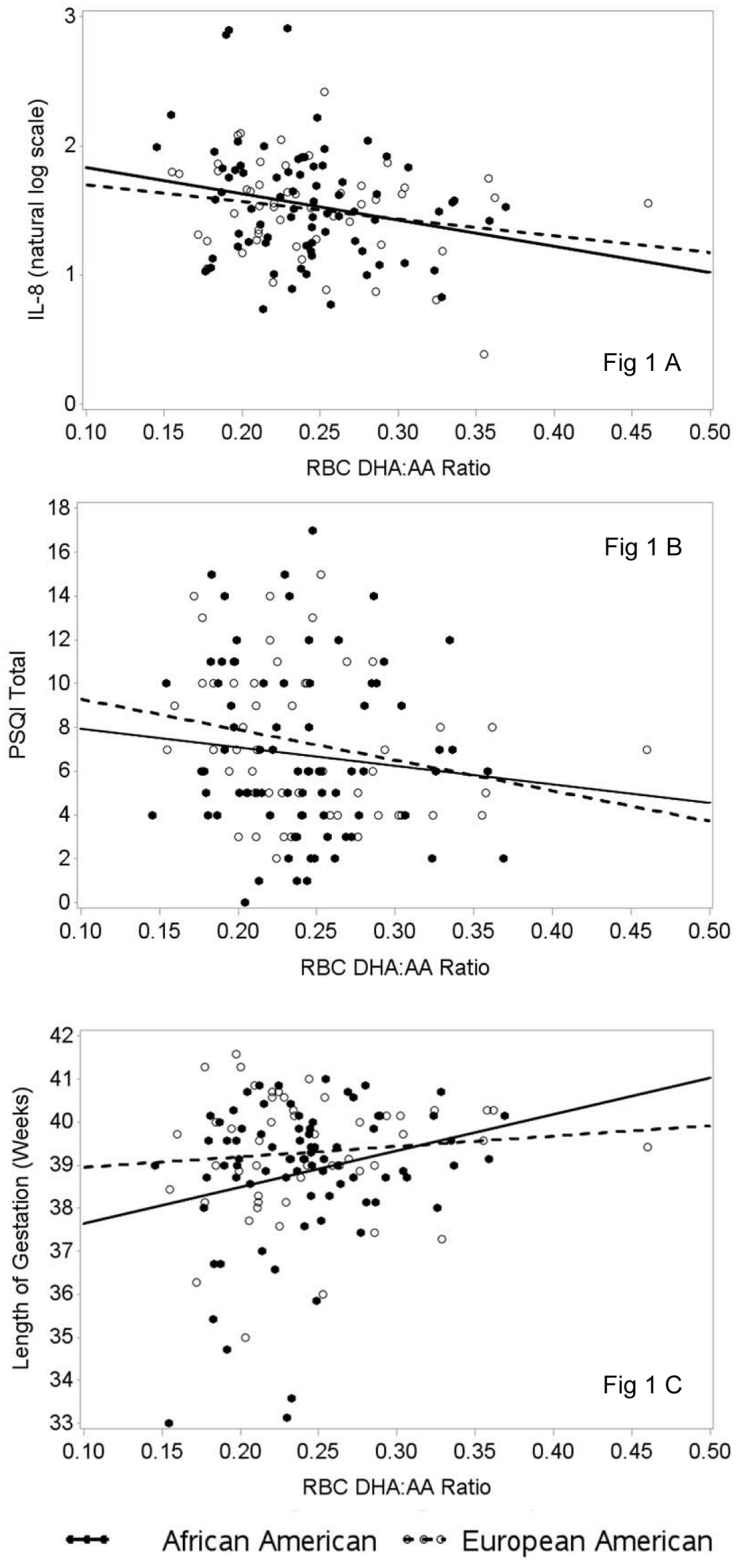
A-C. Red blood cell fatty acid levels, serum IL-8, sleep quality and birth outcomes. **A)** The DHA:AA ratio was significantly associated with serum IL-8 (r = -0.20, p = 0.02). No interactions by race were observed. **B)** Higher RBC DHA:AA ratios predicted better sleep quality, including after adjustment for depressive symptoms, age, BMI, income, race, and smoking, (b = -18.4, p < 0.001). No interactions by race were observed. **C)** A significant interaction between race and the DHA:AA ratio was observed in predicting PTB (p = 0.049). Among African Americans, odds of PTB decreased as DHA:AA increased (OR for 0.1 unit increase = 0.25 (95% CI = 0.06, 0.99), p = 0.048). This association was not present among European Americans (p = 0.99).

RBC DHA levels were not associated with serum IL-6, IL-8, IL-1β, TNF-α, CRP (ps > 0.12), nor was RBC EPA (ps > 0.44). Adjusting for the a prior specified covariates (age, BMI, depressive symptoms, smoking status, and race), higher RBC AA was associated with higher IL-8 (b = 0.07, p = 0.028), as was a lower DHA:AA ratio (b = -1.77, p = 0.018; Fig 1A).

### Inflammation as a Mediator Linking PUFAs and Length of Gestation

In the overall sample, higher IL-8 served as a significant mediator linking higher AA (indirect effect 95% CI = -0.15, -0.01) and a lower DHA:AA ratio (95% CI = 0.10, 3.84) with shorter gestation as a continuous measure. No significant interactions with race were observed.

### RBC Fatty Acids and Sleep Quality

Adjusting for the a priori specified covariates, linear regression analyses demonstrated that higher RBC DHA levels were associated with significantly better overall sleep quality, as indicated by lower total scores on the PSQI (b = -1.00, p = 0.012), as well as subscales indicating longer sleep duration (p = 0.019), and better sleep efficiency (p = 0.047). Neither EPA nor AA was associated with overall sleep quality (ps ≥ 0.34).

Further analyses showed that, adjusting for a priori specified covariates, higher DHA:AA ratios were associated with better overall sleep quality (b = -15.4, p = 0.005; Fig 1B). Moreover, subscale analysis demonstrated that higher DHA:AA ratio was associated with shorter sleep latency (p = 0.033), longer sleep duration (p = 0.019), and better habitual sleep efficiency (p = 0.026). Subsequent analyses utilizing race as an interaction term showed that associations between sleep and fatty acids did not differ significantly by race, as none of the fatty acid by race interactions were significant (*p*s > 0.14).

### Sleep as a Mediator Linking PUFAs and Length of Gestation

In the full sample, overall sleep quality served as a significant mediator linking RBC DHA (95% CI = 0.03, 0.28) and the DHA:AA ratio (95% CI = 0.35, 3.99) with length of gestation. This relationship was not observed in relation to AA (95% CI = -0.08, 0.05). No significant interactions by race were observed in these relationships using length of gestation as a continuous variable.

### Unifying Model Linking RBC Fatty Acids, Sleep, Inflammation, and Length of Gestation

Serial mediation models were fit moving from RBC fatty acids → sleep quality → IL-8 → length of gestation. Models starting with DHA (95% CI = 0.001, 0.06) and the DHA:AA ratio (95% CI = 0.02, 0.79) were statistically significant. The model starting with AA was not significant (95% CI = -0.01, 0.01). The results of these serial mediation models did not differ by race.

### RBC PUFAs and Preterm Birth

In relation to the DHA:AA ratio, a significant interaction with race was observed in predicting preterm birth (p = 0.049; Fig 1C). Among African American women, the odds of preterm birth decreased significantly as DHA:AA increased (OR for 0.1 unit increase = 0.25 (95% CI = 0.06, 0.99), p = 0.048). This association was not present among European American women (p = 0.99).

## Discussion

In this study of 135 pregnant women assessed in mid-gestation, both serum inflammatory markers and sleep quality served as mediators linking RBC PUFA status with length of gestation. Specifically, higher serum IL-8 significantly mediated the association between higher AA and a lower DHA:AA ratio with shorter gestation. In addition, poorer sleep quality, as indicated by higher scores on the PSQI, mediated associations between lower DHA and a lower DHA:AA ratio with shorter gestation. Finally, in serial mediation models, significant paths were found linking both DHA and the DHA:AA ratio with length of gestation via linked effects on sleep quality and inflammation. These associations were not accounted for by concomitant depressive symptoms, or other potential confounders examined, including BMI, smoking, age, and income. Moreover, no effects of EPA were observed in these analyses, indicating the DHA was the critical ω-3. This study provides evidence for novel pathways by which maternal RBC fatty acid status may influence length of gestation.

Ω-3 PUFA supplementation in pregnant women and infants has been examined in relationship to various outcomes including birth weight, length of gestation, food allergy, wheezing, asthma, cognitive development, neurological development, and depression [46]. The dearth of studies examining sleep is notable, particularly given the strong emphasis on potential neurological, cognitive, and mood effects of ω-3 PUFAs in the larger literature. Sleep is a critical aspect of overall health, with direct effects on a myriad of physical and psychological health outcomes.

The current study focused on the clinical implications of fatty acid status for sleep quality and length of gestation. Importantly, maternal sleep is a strong predictor of postpartum maternal mood [47–52]. Among 56 women at risk for depression, every 1 point increase in poor sleep (per PSQI) during the first 17 weeks postpartum was associated with 25% increased risk of PPD [41]. Sleep disruption is also associated with declines in marital satisfaction in the first year postpartum [53]. Thus, if effects of PUFA status on sleep extend to the postpartum period, this may also have clinical implications for maternal mood and related outcomes.

As described, in our prior analyses in the same sample of women, we found that associations between sleep quality, serum IL-8, and length of gestation were driven primarily by effects among African American women [25]. In the current study, associations of RBC fatty acids with sleep quality and IL-8 were similar among African Americans and Whites. In addition, mediation models showed no significant interactions with race in the prediction of length of gestation as a continuous variable. However, an interaction between race and DHA:AA RBC fatty acid levels was observed in the prediction of preterm birth, indicating heightened risk for preterm birth in the context of suboptimal DHA:AA levels in African Americans relative to European Americans. Thus, this presents a pathway which may contribute to persistent racial disparities in preterm birth.

With a preterm birth rate of only 5.7% (n = 3) among Whites in this cohort, our statistical power to detect effects of RBC PUFA status among White women was particularly low; however Black and White women did exhibit similar DHA levels, thus, these data do suggest enhanced vulnerability to PTB in the context of poor DHA status in Blacks. Of note, while preterm babies (born prior to 37 weeks gestation) experience the most severe complications, early term births (37 weeks 0 days– 38 weeks 6 days) are also characterized by increased risk for morbidity and mortality compared to full term births (≥ 39 weeks)[37]. Thus, factors that shift delivery timing even in a relatively subtle manner can have a clinically meaningful impact on neonatal outcomes.

**Fig 2:**
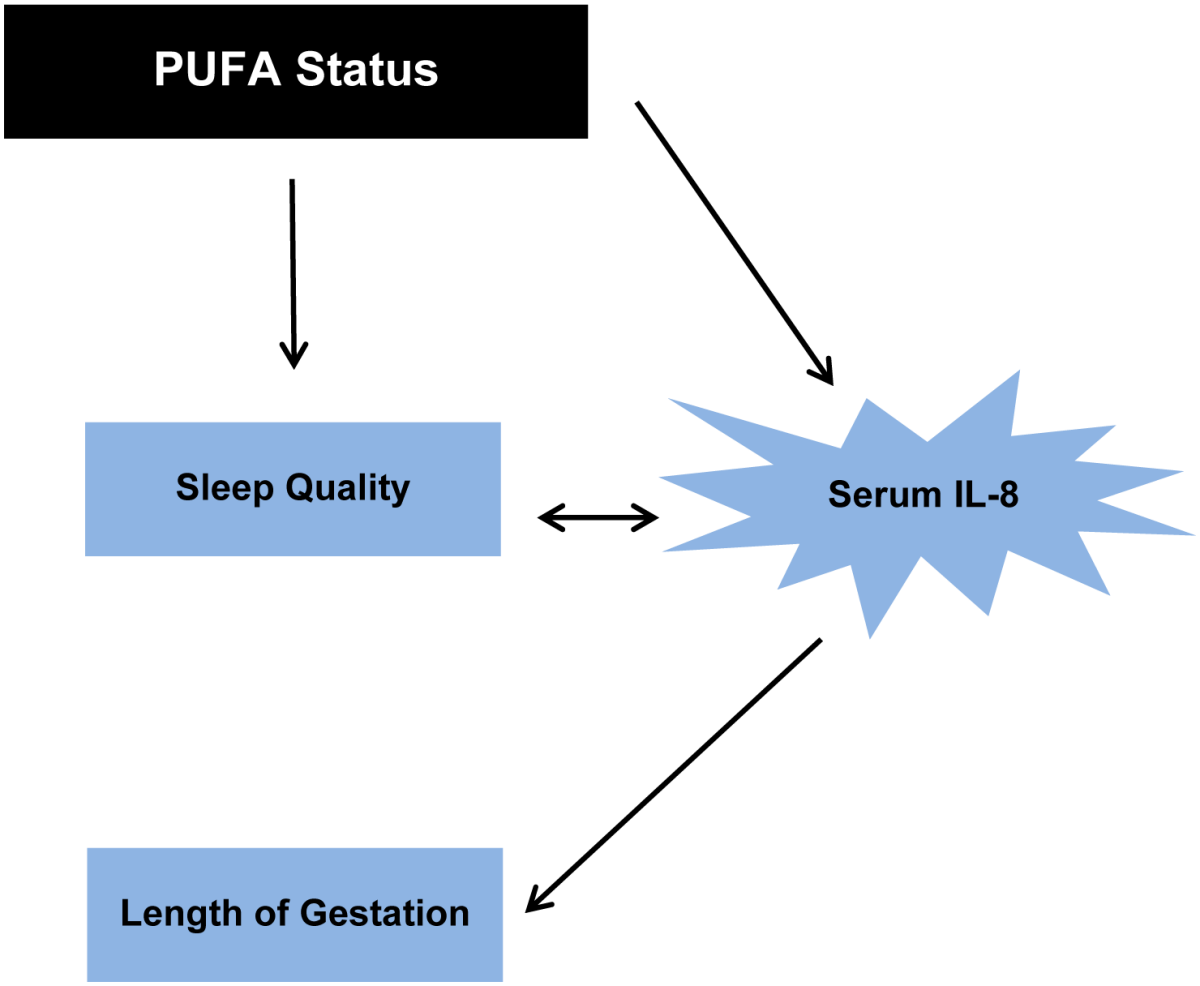
Model Linking Fatty Acid Status, Inflammation, Sleep Quality, and Length of Gestation. Represented by light boxes, we previously found that sleep quality was linked with length of gestation via serum IL-8 (Blair et al., 2015). We now demonstrate a role for fatty acid status within this model. Specifically, serial mediation models moving from RBC fatty acids → sleep → IL-8 → length of gestation demonstrated significant paths starting with DHA (95% CI = 0.001, 0.06) and the DHA:AA ratio (95% CI = 0.02, 0.79).

The observational nature of these data prevents firm delineation of the relative influence of direct and indirect pathways linking LC-PUFAs, sleep quality, and inflammation. Most clearly, as described earlier, sleep quality is linked to inflammation in a bi-directional manner. Our current analyses utilized a causal model from RBC fatty acids → sleep quality → IL-8 → length of gestation (See Fig 2). However, a model in which IL-8 was fitted to precede sleep quality in the causal chain yielded similar results (analyses not shown). It is likely these relationships are bi-directional; suboptimal LC-PUFA consumption may increase inflammation, inducing sleep disturbance which may further drive inflammation.

With regard to the particular relevance of IL-8 in these associations, as described, we previously reported that poor sleep quality was linked to shorter gestation via IL-8 in this cohort [25]. As described in our earlier report, higher IL-8 has also been reported in relation to shorter sleep duration among depressed pregnant women and has repeatedly been observed in relation to obstructive sleep apnea [54, 55]. In pregnancy, elevated serum, intra-amniotic, and cervical IL-8 has been associated with preterm and term parturition [56–59]. As a chemokine, IL-8 is a potent chemoattractant implicated in endothelial dysfunction. As such, IL-8 contributes to atherosclerosis and is indicated in risk of cardiovascular events [60]. Of note, placental blood flow has been implicated in risk for preterm delivery, as has maternal endothelial dysfunction [61, 62]. Thus, vascular effects present a potential mechanistic link by which elevated IL-8 may affect birth timing.

Although anti-inflammatory effects of ω-3 PUFAs are well-established in preclinical studies, the clinical literature is mixed and characterized by frequent null findings [63]. Contributing to inconsistencies is considerable heterogeneity in sample size, study populations (diseased versus healthy), design (observational versus interventional), and dosage (among intervention studies). Beneficial effects of dietary ω-3 have most commonly been observed in relation to IL-6, TNF-α, and CRP [64–66]. Moreover, these effects are seen more frequently in diseased rather than healthy populations [64, 65], plausibly due to generally low levels of inflammatory markers in healthy groups, resulting in a floor effect. Given that pregnancy is characterized by elevations in serum inflammatory markers [67], effects of fatty acid status on inflammation may be more readily observable than in healthy non-pregnant adults.

IL-8 has less commonly been included as an outcome marker. However, effects of ω-3 PUFAs on circulating IL-8 levels as well as stimulated IL-8 production have been reported [66, 68–70]. Of relevance, one study examined effects of ω-3 PUFAs on inflammatory responses of the amnion, the membrane forming the amniotic sac. Using samples collected via ceasarean deliveries, samples cultured with DHA or DHA+EPA exhibited significantly reduced IL-8 as well as IL-6 secretion [71]. Moreover, in an RCT of overweight/obese pregnant women, women randomized to receive DHA+EPA (2g/day) showed reduction in plasma CRP, but not IL-8 [66]. However, supplemented women had significantly lower expression of IL-8 and other inflammatory markers in adipose and placental tissue compared with the placebo group [66]. The current study did not include assessment of these types of localized effects, which may ultimately have greater predictive value for birth outcomes.

The lack of predictive value for other inflammatory markers within this model is notable. In particular, serum, cervical, and intra-amniotic IL-6 has been linked with preterm birth risk [72–74] as well as sleep quality [75]. Moreover, DHA supplementation has been observed to reduce serum levels of IL-6 as well as other inflammatory markers [76]. Replication studies are required to determine if additional markers emerge as predictive within similar models in larger samples inclusive of greater diversity in terms of SES, fatty acid intake, and sleep quality parameters. In addition, effects may be more apparent in a clinically disordered population (e.g., preeclampsia) in which dysregulation of inflammatory processes is apparent.

It is notable that prior studies have similarly found that higher DHA in particular is associated with better sleep quality [31, 32, 77]. For example, among 362 children ages 7–9, poorer sleep quality was modestly associated with lower serum DHA and a lower DHA:AA ratio [77]. Subsequently, following a 16-week supplementation with DHA versus placebo, actigraphy performed in a random subset of 43 children demonstrated considerably fewer waking episodes and longer total sleep duration in DHA supplemented children. These results are highly consistent with the current study, in which analyses of PSQI subscales indicated that higher DHA and higher DHA:AA ratios were associated with longer sleep duration and better sleep efficiency as well as shorter sleep latency. Our current analyses were hypothesis-driven based on the existing literature supporting effects of DHA on sleep. However, full RBC fatty acid profiles were obtained, see S1 Table. Secondary analyses showed no predictive value for other fatty acids for either sleep quality or length of gestation in the overall cohort or in African American and European American women assessed separately (analyses not shown).

The current study focused on lower income women. Thus, generalizability to other populations is unknown. Surprisingly, the overall levels of RBC fatty acids were not markedly lower than observed in other US cohorts of higher socioeconomic status. For example, the mean DHA level in this sample was 3.5%, compared to 3.7% in a study of 306 women ages 30–55 years who participated in the Nurses’ Health Study [78]. This is unexpected given the demands of pregnancy as well as poorer dietary quality associated with lower socioeconomic status. However, regardless of socioeconomic status, DHA consumption in women in North America is low [79]. The American Academy of Pediatrics and other expert groups recommend that pregnant and lactating women consume 200–300 mg/day of DHA [80]. This is well above the estimated ~80 mg/day consumed by North American women during pregnancy [81], and markedly above the estimated 48 mg/day that has been reported among lower income pregnant women in the US [82]. Given the marked difference between actual and recommended intake, supplementation may be the most effective solution for increasing consumption in pregnant women.

Sleep quality among African Americans and European Americans was highly similar in this sample. Prior studies demonstrate racial disparities in sleep, even after accounting for socioeconomic status [83, 84]. It is likely that such racial differences are more readily observable in studies inclusive of greater socioeconomic diversity; compared to Whites, African Americans may not experience the same degree of improvement in sleep with increasing socioeconomic status [85]. Given that this sample was predominately of lower SES, racial differences in the health-related burden of poor sleep are likely underestimated.

A limitation of this study was that it was cross-sectional in design; the PSQI assesses sleep quality over the past month. Given known longitudinal changes in fatty acid status as well as sleep parameters, it would be useful to examine the association between these factors over time. It would also be informative to determine whether the observed associations between fatty acid status and subjective sleep quality are also present in relation to sleep quality measured using objective indicators. In addition, this study included multiple comparisons without statistical adjustment. The observed associations between DHA and sleep are as hypothesized and consistent with prior studies. However, replication in additional cohorts would increase confidence in results.

A strength of the current study is the utilization of RBC fatty acid levels. Although many prior studies have utilized serum or plasma measures of fatty acids, RBC fatty acid levels show greater temporal stability and may be more highly correlated with measured dietary intake.[78]

Despite strong correspondence between RBC fatty acid levels and long-term fatty acid intake as measured by food-frequency questionnaire (FFQ) [78], we did not assess dietary fat intake in the current studies. A prior study concluded that for every gram of daily dietary intake, RBC concentrations of long-chain ω-3 PUFAS increased by only 1.7% in Black women compared to 4.8% in Whites [86]. Notably, data show that maternal genetic variants in the fatty acid desaturase gene (FADS) cluster modify the association between maternal LC-PUFA intake and birth weight [87]. Moreover, evidence suggests that Blacks show greater occurrence of FADS genes associated with elevated arachidonic acid which may reduce ω-3 synthesis due to competitive metabolic pathways [88, 89]. Thus, assessment of dietary intake as well as genetic markers would help to explicate the role of consumption versus metabolic processes in determining RBC fatty acid status. Data on diet would also provide information regarding the overall nutritional status of the women; it is possible that other dietary factors co-vary with DHA intake and contribute to observed effects.

In addition, longitudinal assessment across pregnancy would provide valuable insight into the temporal dynamics of RBC fatty acids and sleep, both of which show marked changes across the course of gestation. In particular, both sleep quality and ω-3 fatty acid levels decline in the 3^rd^ trimester of pregnancy. A particular focus on this vulnerable period would be of value in future studies.

Prior studies demonstrate that increased fatty acid intake via supplements or food can result in marked and stable improvements in serum DHA within one month of initiation and prevent pregnancy-related declines in maternal levels [1, 90]. Reflecting recognition of the importance of both DHA and AA to infant development, these are supplied together in infant formulas. However, there are many unanswered questions with regard to the extent to which both of these nutrients are needed, as well as the ideal balance with which they should be supplied. Although benefits of DHA consumption in pregnant women and infants are well-documented, few studies have found any additional benefit of DHA+AA compared to DHA alone [14]. In addition, as noted earlier, human and animal studies demonstrate that whereas DHA levels in breast milk and brain are highly dependent on diet, AA levels are less affected by intake, but instead influenced greatly by genetic factors. Given this evidence, it has been suggested that compared to the clinical effects of dietary DHA in the prenatal period and early life, dietary AA may play only a minor role in growth and development [14]. Results of the current study are consistent with the notion that dietary DHA is of particular value.

In sum, the current study supports mediating roles for inflammation and poor sleep in linking poorer RBC fatty acid status with shorter gestation, with greater risk of preterm birth among African Americans versus European Americans in the context of a low DHA:AA ratio. Continued attention to potential racial differences in the risk for adverse perinatal health sequelae of suboptimal LC-PUFA intake is warranted. Adequate representation of African American women and examination of potential effects in subgroups may reveal clinically meaningful effects and enhance consistency of findings across studies.

## Supporting Information

S1 Dataset Limited dataset including key variables in analyses presented. PSQI = Pittsburgh Sleep Quality Index; age = Maternal age; prepregnancyBMI = Body Mass Index (kg/m^2^) prior to the current pregnancy; gest_age_birth_days = gestational age at birth in days; race: 1 = African American, 2 = European American, 99 = Other; log_IL8 = log 10 transformed serum interleukin-8. (SAV)

S1 Table Complete RBC Fatty Acid levels by Race. Values are expressed as mg/100 mg of total fatty acids identified: mean (SD). (DOCX)

## Abbreviations

DHA: docosahexaenoic acid
AA: arachidonic acid
LC-PUFA: long-chain polyunsaturated fatty acids
RBC: red blood cell
PSQI: Pittsburgh Sleep Quality Index
PTB: preterm birth
TNF-α: tumor necrosis factor-α
IL: interleukin
CRP: C-reactive protein
CES-D: Center for Epidemiologic Studies Depression Scale
