# Supplementary material for: Polyunsaturated Fatty Acid (PUFA) Status in Pregnant Women: Associations with Sleep Quality, Inflammation, and Length of Gestation
Source: PLoS One. 2016 Feb 9;11(2):e0148752. doi: 10.1371/journal.pone.0148752 (PMC4747600; doi:10.1371/journal.pone.0148752)
Supplement: S1 Table — (DOCX) [file pone.0148752.s002.docx]

**Supplemental Table: Complete RBC Fatty Acid levels by Race**

|  | **Total (n=135)** | **African-American (n=78)** | **European American (n=51)** | **Group comparison (p value)** |
| --- | --- | --- | --- | --- |
| 14:0 (myristic) | 0.51 (0.18) | 0.46 (0.15) | 0.59 (0.18) | <.0001 |
|  | 0.2-1.1 | 0.2-1.0 | 0.3-1.1 |  |
| 16:0 (palmitic) | 26.0 (1.4) | 25.9 (1.4) | 26.2 (1.4) | 0.26 |
|  | 22.2-29.9 | 22.2-29.4 | 22.8-29.9 |  |
| 16:1n7 (palmitoleic) | 1.0 (0.35) | 0.87 (0.26) | 1.2 (0.39) | <.0001 |
|  | 0.4-2.4 | 0.4-1.6 | 0.5-2.4 |  |
| 18:0 (stearic) | 16.5 (1.3) | 16.8 (1.3) | 16.1 (1.2) | 0.0043 |
|  | 12.3-20.5 | 13.3-20.5 | 12.3-18.5 |  |
| 18:1n9 (oleic) | 15.6 (2.2) | 12.9 (0.97) | 13.7 (0.87) | <.0001 |
|  | 10.2-16.2 | 10.2-15.1 | 12.0-16.2 |  |
| 18:2n6 (linoleic) | 15.6 (2.2) | 15.6 (2.2) | 15.5 (2.2) | 0.92 |
|  | 11.4-24.1 | 11.5-24.1 | 11.4-22.7 |  |
| 18:3n3 (alpha-linolenic) | 0.3 (0.1) | 0.28 (0.1) | 0.31 (0.1) | 0.08 |
|  | 0.1-0.6 | 0.1-0.6 | 0.2-0.6 |  |
| 20:2n6 (eicosadienoic) | 0.6 (0.2) | 0.6 (0.1) | 0.7 (0.3) | 0.046 |
|  | 0.4-1.8 | 0.4-1.3 | 0.4-1.8 |  |
| 20:3n6 (dihomo-gamma-linolenic) | 2.0 (0.5) | 1.8 (0.4) | 2.2 (0.5) | <0.001 |
|  | 1.3-3.7 | 1.3-3.6 | 1.4-3.7 |  |
| 20:4n6 (arachidonic) | 14.5 (1.3) | 14.9 (1.2) | 14.0 (1.2) | <0.001 |
|  | 10.5-17.7 | 12.2-17.7 | 10.5-16.7 |  |
| 20:5n3 (eicosapentaenoic) | 0.2 (0.1) | 0.2 (0.1) | 0.2 (0.1) | 0.45 |
|  | 0.1-0.7 | 0.1-0.7 | 0.1-0.5 |  |
| 22:4n6 (adrenic) | 3.8 (0.8) | 3.9 (0.7) | 3.7 (0.8) | 0.12 |
|  | 1.5-5.7 | 1.9-5.7 | 1.5-5.0 |  |
| 22:5n3 (docosapentaenoic) | 1.6 (0.3) | 1.6 (0.3) | 1.6 (0.3) | 0.94 |
|  | 0.9-2.5 | 0.9-2.5 | 0.9-2.0 |  |
| 22:5n6 (docosapentaenoic) | 0.7 (0.2) | 0.7 (0.2) | 0.6 (0.2) | 0.78 |
|  | 0.2-1.3 | 0.3-1.3 | 0.3-1.2 |  |
| 22:6n3 (docosahexaenoic) | 3.5 (0.7) | 3.6 (0.7) | 3.4 (0.8) | 0.20 |
|  | 1.9-5.8 | 2.2-5.6 | 1.9-5.8 |  |

***Mean (SD) and range***

**Values are expressed as mg/100 mg of total fatty acids identified: mean (SD)**
